# Janus Compounds, 5-Chloro-*N*^4^-methyl-*N*^4^-aryl-9*H*-pyrimido[4,5-*b*]indole-2,4-diamines, Cause Both Microtubule Depolymerizing and Stabilizing Effects

**DOI:** 10.3390/molecules21121661

**Published:** 2016-12-02

**Authors:** Cristina C. Rohena, April L. Risinger, Ravi Kumar Vyas Devambatla, Nicholas F. Dybdal-Hargreaves, Roma Kaul, Shruti Choudhary, Aleem Gangjee, Susan L. Mooberry

**Affiliations:** 1 Department of Pharmacology, University of Texas Health Science Center at San Antonio, San Antonio, TX 78229, USA; crohena@ucsd.edu (C.C.R.); risingera@uthscsa.edu (A.L.R.); hargreavesnd@livemail.uthscsa.edu (N.F.D.-H.); kaulr@livemail.uthscsa.edu (R.K.); 2 Cancer Therapy & Research Center, San Antonio, TX 78229, USA; 3 Division of Medicinal Chemistry, Graduate School of Pharmaceutical Sciences, Duquesne University, Pittsburgh, PA 15282, USA; drkvyas@gmail.com (R.K.V.D.); choudharys@duq.edu (S.C.)

**Keywords:** microtubule, microtubule stabilizer, microtubule destabilizer, cancer, colchicine site

## Abstract

While evaluating a large library of compounds designed to inhibit microtubule polymerization, we identified four compounds that have unique effects on microtubules. These compounds cause mixed effects reminiscent of both microtubule depolymerizers and stabilizers. Immunofluorescence evaluations showed that each compound initially caused microtubule depolymerization and, surprisingly, with higher concentrations, microtubule bundles were also observed. There were subtle differences in the propensity to cause these competing effects among the compounds with a continuum of stabilizing and destabilizing effects. Tubulin polymerization experiments confirmed the differential effects and, while each of the compounds increased the initial rate of tubulin polymerization at high concentrations, total tubulin polymer was not enhanced at equilibrium, likely because of the dueling depolymerization effects. Modeling studies predict that the compounds bind to tubulin within the colchicine site and confirm that there are differences in their potential interactions that might underlie their distinct effects on microtubules. Due to their dual properties of microtubule stabilization and destabilization, we propose the name Janus for these compounds after the two-faced Roman god. The identification of synthetically tractable, small molecules that elicit microtubule stabilizing effects is a significant finding with the potential to identify new mechanisms of microtubule stabilization.

## 1. Introduction

Compounds that target microtubules have been instrumental as biological probes to identify the nature of tubulin and the role of tubulin dynamics in mitosis and more recently, in interphase. Microtubule targeting agents have a long and storied history of utility in the treatment of cancer and new and improved agents continue to be approved for the treatment of a wide variety of tumors. Compounds that disrupt microtubules are often divided in two groups. Those that stimulate tubulin polymerization and increase the density of cellular microtubules are referred to as microtubule stabilizers, which include the taxanes, epothilones, laulimalide, peloruside A, zampanolide and the taccalonolides [1,2,3]. In contrast, microtubule depolymerizers or destabilizers cause the loss of cellular microtubules and inhibit tubulin polymerization. Members of this class include the vinca alkaloids, colchicine and combretastatin A-4 (CA-4), halichondrin B, maytansine, the cryptophycins, eribulin and numerous other natural and synthetic compounds [2,4,5,6,7,8]. The clinical successes of a number of microtubule stabilizers and destabilizers, together with their broad spectrum of anticancer actions has fueled the continued search for new compounds that can overcome some of the limitations of currently approved microtubule targeting drugs, including the ability to overcome multidrug resistance mechanisms.

A goal of our research is to identify synthetically tractable microtubule disrupting compounds with better aqueous solubility and the ability to circumvent P-glycoprotein (Pgp) and βIII tubulin-mediated drug resistance. We have identified multiple compounds with potent antiproliferative effects that bind within the colchicine site on tubulin and have in vivo antitumor activities [9,10,11,12,13,14,15]. While evaluating a new series of compounds, we identified four that have unique effects on cellular microtubules. These compounds initially demonstrated classic microtubule destabilizing effects, but at higher concentrations they caused distinct effects reminiscent of microtubule stabilization. Additionally, there are differences among the four compounds in their propensities to cause these dual effects. Detailed experiments were conducted to further investigate the effects of these compounds in cells and with purified tubulin. The antiproliferative and cytotoxic activities of the compounds against a range of cancer cell lines were evaluated, including their efficacy in multidrug resistant cell lines. Molecular modeling was used to predict interaction between the compounds and the colchicine site on tubulin. This group of compounds is unique in that they cause distinct microtubule stabilizing and destabilizing effects and our results suggest they have the potential to serve as useful tools for the study of microtubule-drug interactions.

## 2. Results

### 2.1. Chemical Synthesis

**Scheme 1:**
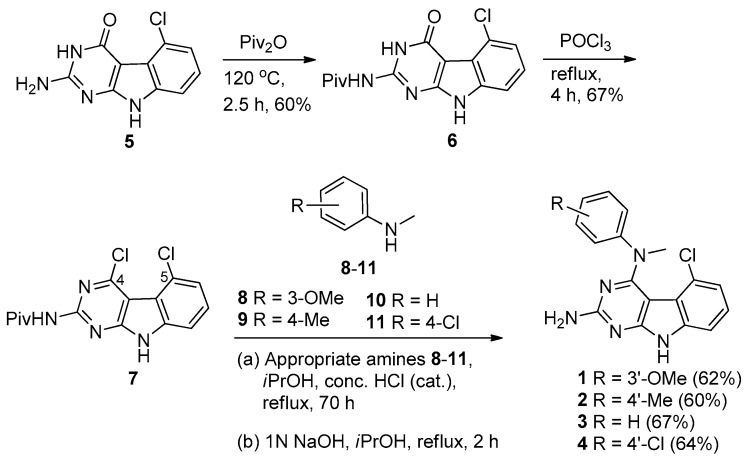
Synthesis of target compounds **1**–**4**.

**Figure 1:**
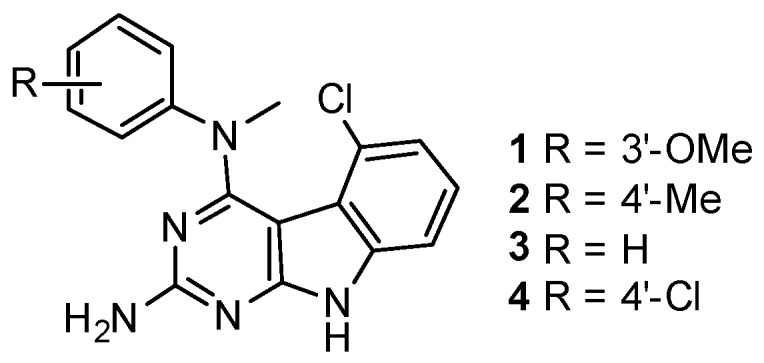
Chemical structures of the Janus compounds **1**–**4**.

The synthesis of compounds **1**–**4** is shown in Scheme 1. Protection of the 2-amino group of **5** using pivalic anhydride provided **6** in 60% yield, better than the reported method [16] which utilized pivalic anhydride, 4-(*N*,*N*-dimethylamino)pyridine (DMAP) and triethylamine to give **6** in 40% yield. The pivaloyl protection of the 2-amino group increased the solubility of the compound in organic solvents for the chlorination step; a direct chlorination of unprotected **5** with phosphorus oxychloride resulted in no reaction. Compound **6** was treated with phosphorus oxychloride to obtain **7** in 67% yield [15]. Displacement of the 4-chloro group in **7** with *N*-methyl anilines **8**–**11** followed by base-mediated deprotection of the 2-amino group provided target compounds **1**–**4**, respectively, in 60%–67% yield. The chemical structures of these compounds are shown in Figure 1.

### 2.2. Effects of Compounds ***1**–**4*** on Cellular Microtubules

**Figure 2:**
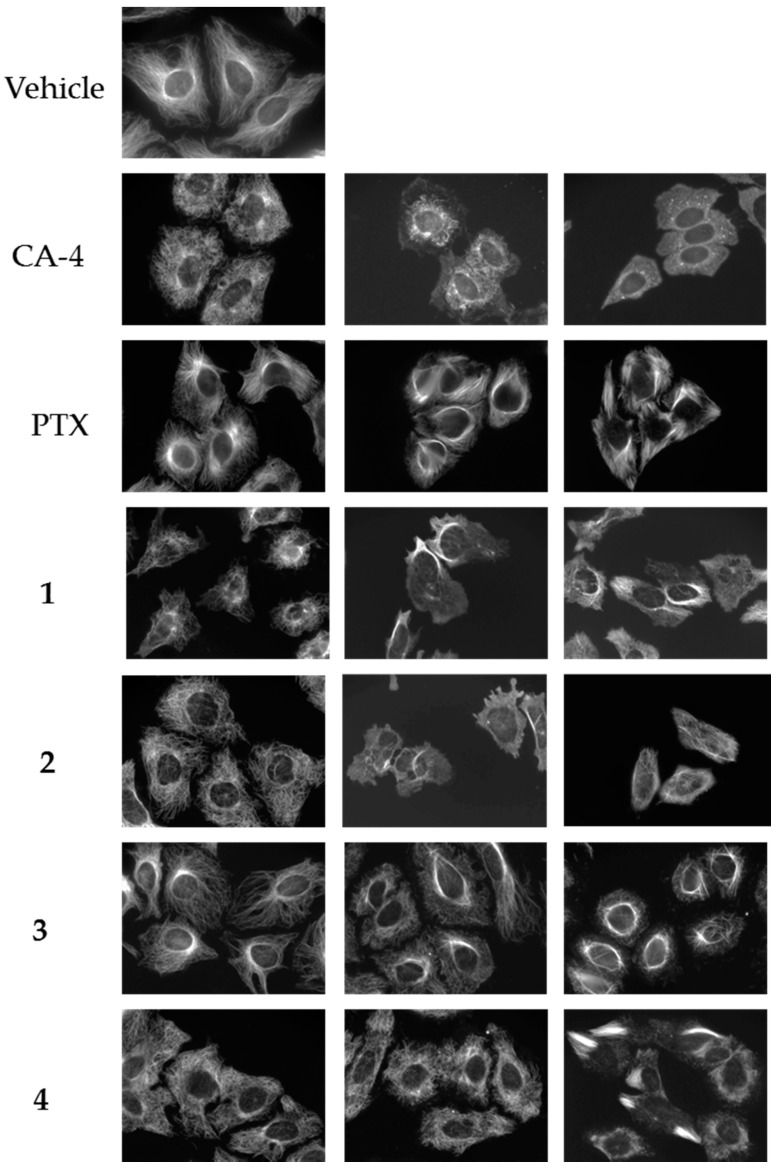
Concentration-dependent effects of the Janus compounds on cellular microtubules. HeLa cells were treated for 18 h with a range of concentrations of the microtubule destabilizer CA-4 (5, 7.5, and 15 nM), the microtubule stabilizer paclitaxel (PTX) (0.1, 0.5, and 1 μM) or compounds **1**–**4**: **1** (30, 50, and 60 μM); **2** (25, 60, and 75 μM); **3** (10, 20, and 30 μM); and **4** (20, 30, and 35 μM). Microtubules were visualized by indirect immunofluorescence.

Four compounds (**1**–**4**) with unique effects on microtubules were identified while screening a library of synthetic compounds for microtubule disrupting activities. Each of these compounds was found to cause alterations in microtubule structure consistent with microtubule depolymerization at 10–25 μM. However, in contrast to the progressive microtubule loss that was expected to be observed with classic microtubule destabilizing drugs, bundles of microtubules were observed as concentrations were increased. The effects of these compounds on cellular microtubules were investigated further using a wide range of concentrations. The results show that compounds **1**–**4** have concentration-dependent microtubule depolymerizing and stabilizing effects and that there are differences among the compounds (Figure 2).

The normal microtubule array in HeLa cells consists of microtubules emanating from the central region of the cell, the location of the microtubule organizing center, and extending to the cell periphery (Figure 2, vehicle). The concentration dependent effects of the classic microtubule destabilizer combretastatin A-4 (CA-4) are shown on the next line (Figure 2, CA-4). At the lowest concentrations that affect interphase microtubule structures, 5 nM here, microtubules took on a less rigid, slightly disorganized morphology that we will refer to as relaxed (CA-4, left panel). At a slightly higher (7.5 nM) concentration of CA-4, there was a progressive loss of microtubules from the cell periphery observed with a collapse of the remaining microtubules around the nucleus (CA-4, center panel). At 15 nM CA-4, a total loss of cellular microtubules was observed (CA-4, right panel) with a few remnant microtubules remaining in the cytoplasm. In contrast, the microtubule stabilizer paclitaxel caused a slight increase in the density and rigidity of microtubules throughout the cytoplasm at 100 nM (PTX, left panel) and, at 500 nM, diffuse microtubule bundles were primarily localized around the nucleus (PTX, center panel). A higher concentration of 1 µM paclitaxel resulted in thick, rigid, discrete microtubule bundles that were localized throughout the cell (PTX, right panel).

**Figure 3:**
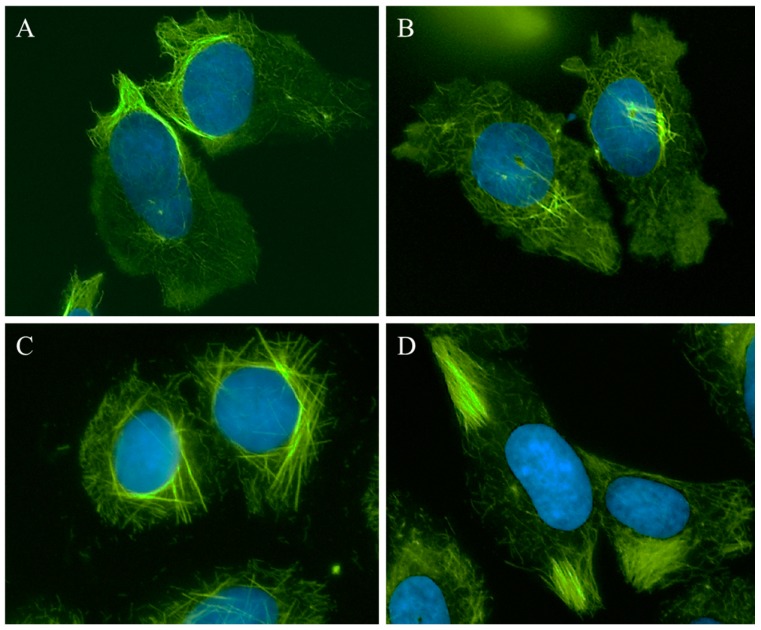
Microtubule stabilization and destabilization elicited by Janus compounds. HeLa cells were treated with: (**A**) 50 μM **1**; (**B**) 60 μM **2**; (**C**) 30 μM **3**; or (**D**) 35 μM **4** for 18 h. Microtubules were visualized by indirect immunofluorescence (green) and DNA with DAPI (blue).

A 25 µM concentration of **1** caused the appearance of relaxed microtubules, similar to the lowest concentrations of CA-4 and more peripheral depolymerization was observed at 30 µM (Figure 2, **1**, left panel). At 40–50 µM**,** the predominant effect of **1** was microtubule loss; however, some bright tubulin polymers were also visible in many cells, suggestive of microtubule stabilization. Often clear microtubule depolymerizing and stabilizing effects were visible in the same cell, with areas of complete microtubule loss alongside bundles of microtubules (Figure 3A). At 50–60 µM, the mixed effects of **1** were evident with dense microtubule bundles adjacent to regions of microtubule depolymerization (**1**, center and right panels). Compound **2** showed similar concentration-dependent microtubule depolymerizing effects with relaxed, disorganized microtubules observed at 25 µM (**2**, left panel) and more extensive microtubule depolymerization was observed at 30 µM (data not shown). Between 40 and 60 µM, microtubule depolymerization continued to be observed, but was often accompanied by bright tubulin polymers (**2**, center panel; Figure 3B). At 75 µM, rigid microtubule structures, reminiscent of the effects of high concentrations of paclitaxel, but markedly distinct in structure in that they are less tightly bundled and less abundant, were found localized throughout some cells (Figure 2, **2**, right panel). Thus, for both **1** and **2**, concentrations of 25–40 µM resulted in microtubule destabilizing effects that were similar to those observed at 5 and 7.5 nM CA-4. However, as the concentrations were increased, bright, rigid tubulin structures and areas of dense microtubules were apparent in cells that also had clear microtubule destabilization (Figure 3A,B). This phenotype of mixed microtubule stabilizing and destabilizing effects was not seen at any concentration with the classic microtubule stabilizing or destabilizing drugs paclitaxel or CA-4.

Compounds **3** and **4** also demonstrated mixed effects on cellular microtubule structures, although only modest microtubule depolymerizing effects were observed before the stabilizing effects of these two compounds dominated. At a concentration of 10 μM, **3** caused some slight microtubule relaxation, although cells looked similar to vehicle-treated controls (Figure 2, **3**, left panel). At 20 μM, mixed effects were observed with the microtubules in the periphery of the cell demonstrating a more relaxed, disorganized morphology with more bundled microtubule structures localized around the nucleus (**3**, center panel). At 30 µM, **3** caused the appearance of discrete, needle-like microtubule bundles around the nucleus accompanied by a lack of microtubules in the cell periphery (**3**, right panel; Figure 3C). Similarly, 20–30 µM concentrations of **4** caused modest microtubule depolymerizing effects with relaxed microtubules that were similar to the effects of 5 nM CA-4 (**4**, left and center panels). Consistent with the effects of the other Janus compounds, a 35 µM concentration of **4** caused the formation of discrete microtubule bundles that, in this case, were most prevalent in the cell periphery (Figure 3D). The bundling of cellular microtubules that occurred with 35 µM **4** was more reminiscent of the microtubule bundles elicited by paclitaxel.

While each of the Janus compounds displayed phenotypes associated with both microtubule destabilizers (relaxed, disorganized and depolymerized microtubules) and stabilizers (rigid, bundled microtubules), there were clear differences among them. Compounds **1** and **2** predominantly demonstrated microtubule destabilizing effects with clear microtubule loss observed over a large range in concentrations. However, these compounds did not behave like classic destabilizing agents because at higher concentrations the microtubule destabilization was accompanied by bundled tubulin structures (Figure 3A,B). In contrast, compounds **3** and **4** have more predominant microtubule stabilizing effects, but differ from the effects of paclitaxel in that they initiate microtubule depolymerization prior to, and sometimes alongside, the bundled microtubules. These dual effects of microtubule stabilization and destabilization by the same compound are unanticipated and were not observed for any of the other structurally similar small molecules previously evaluated by our group [9,10,11,12,13,14,15,17]. The fact that these two opposing effects on microtubule structures can be observed in the same cells led us to propose naming these compounds after Janus, the two-faced Roman god.

### 2.3. Effects of Janus Compounds on Tubulin Polymerization

**Figure 4:**
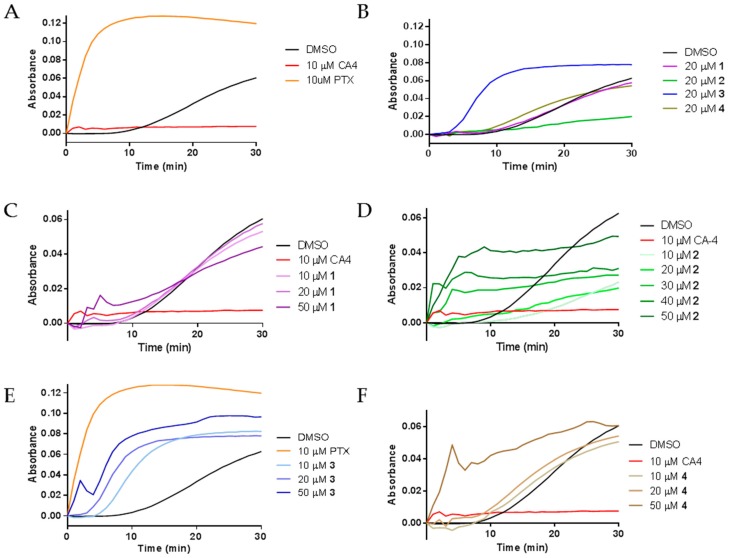
The effects of **1**–**4** on tubulin polymerization: (**A**) the polymerization of purified porcine brain tubulin was monitored by absorbance at 340 nm after incubation with vehicle (DMSO), 10 μM CA-4, or 10 μM paclitaxel (PTX); (**B**) effects of **1**–**4** on tubulin polymerization at stoichiometric concentrations with tubulin; and (**C**–**F**) concentration-dependent effects of **1**–**4** on tubulin polymerization.

The diverse effects of compounds **1**–**4** on interphase microtubules led us to evaluate their activities directly on tubulin by performing purified tubulin polymerization assays. The effects of the compounds on the assembly of purified porcine brain tubulin were evaluated turbidimetrically. As expected, the classic microtubule stabilizer paclitaxel enhanced the rate and extent of tubulin polymerization as compared to the DMSO control while the destabilizer CA-4 inhibited the polymerization of purified tubulin (Figure 4A). The effects of compounds **1**–**4** on purified tubulin polymerization were initially evaluated at concentrations equimolar to tubulin, 20 μM. Compounds **1** and **4** had no effects on tubulin polymerization at 20 µM, as evidenced by the lines overlapping with the vehicle control. Very different effects were seen with compounds **2** and **3**. A 20 µM concentration of **2** inhibited tubulin polymerization, while 20 µM **3** increased the rate and extent of tubulin polymerization (Figure 4B). These tubulin polymerization results are consistent with the effects of the compounds in cells, where the predominant effect of **3** was the formation of tubulin bundles while the most notable effect of **2** was microtubule loss (Figure 2 and Figure 3).

Further tubulin polymerization experiments were conducted to evaluate the concentration dependent effects of each compound on tubulin polymerization. Figure 4C–F shows the concentration dependent effects of compounds **1**–**4**, respectively. Tubulin polymerization was inhibited by 10 and 20 µM **2** (Figure 4D). However, at 30–50 µM, concentration-dependent increases in the initial rate of tubulin polymerization were measured (Figure 4D). While the total levels of tubulin polymer were higher at 50 µM than with lower concentrations, the total tubulin polymer formed in all conditions was less than the vehicle control. The mixed effects on cellular microtubules observed by immunofluorescence suggest that the rapid increase in the initial rate of polymerization without an increase in the total amount of polymer at steady state is likely due to the competing microtubule depolymerizing and stabilizing effects of these drugs. Compound **3** caused a concentration dependent increase in the rate of tubulin polymerization, one characteristic of microtubule stabilizers. A super-stoichiometric, 50 µM, concentration of **3**, did increase both the rate and extent of tubulin polymer formed, but not to the extent seen with a sub-stoichiometric concentration of paclitaxel. These results are consistent with the effects of **3** in cells and suggest that **3** does have microtubule stabilizing activity but with features that are quite different from paclitaxel on both cellular microtubules and with purified tubulin. Tubulin polymerization experiments with 50 µM **1** and **4** also confirmed their ability to increase the initial rate of tubulin polymerization at concentrations super-stoichiometric to tubulin and were consistent with the cellular results where **4** appeared to have more effective microtubule stabilizing effects than **1** (Figure 4C,F). Together, these results demonstrate that all four compounds can directly affect the polymerization of purified tubulin with differential propensity for microtubule stabilizing and destabilizing effects.

**Figure 5:**
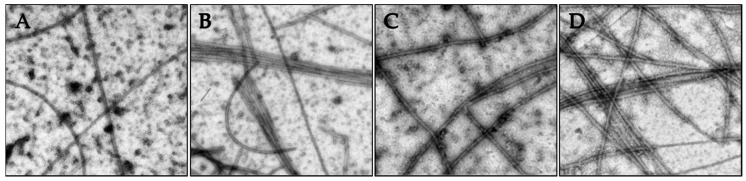
The effects of the Janus compounds on tubulin polymer were visualized by electron microscopy and images shown at 50,000× magnification: (**A**) vehicle control; (**B**) 10 µM paclitaxel; (**C**) 50 µM **2**; and (**D**) 10 µM **3**. The samples were fixed for microscopy following 35 min of polymerization.

The nature of the tubulin polymers formed in the turbidity experiments were evaluated by electron microscopy to specifically investigate whether concentrations of compounds that caused increased turbidity corresponded to the formation of microtubules or to abnormal tubulin aggregates or paracrystals. The vinca alkaloids are known to induce tubulin paracrystals at super-stoichiometric concentrations [18]. As shown in Figure 5, the tubulin polymerization observed in Figure 4 in the presence of vehicle, paclitaxel, **2** or **3** led to the generation of microtubule polymers.

### 2.4. Effects of Janus Compounds on Drug Sensitive and Multidrug Resistant Cancer Cell Lines

Microtubule targeting agents are known to inhibit cellular proliferation and induce cytotoxicity in cancer cells. Therefore, the Janus compounds were evaluated in a panel of drug sensitive and multidrug resistant cancer cell lines. The results show that these compounds have efficacy in cell lines from diverse tumor types with potencies in the low micromolar range, an order of magnitude less potent than paclitaxel or CA-4 (Table 1). Compound **1** was slightly less potent in four of the five cell lines and **2** was slightly more potent in most of the cell lines. The concentrations of compounds **1**–**4** that elicited notable changes in microtubule structure in Figure 2 were determined to be 6–21-fold greater than their respective antiproliferative efficacies in Table 1. This is consistent with the fact that the control microtubule destabilizer, CA-4, and stabilizer, paclitaxel, alter the structure of interphase microtubules at 2- and 36-fold their respective IC_50_ values. Isogenic cell line pairs were used to evaluate the ability of these compounds to overcome P-glycoprotein (Pgp) and βIII tubulin-mediated drug resistance. Consistent with the effects of the positive control CA-4, each of the Janus compounds had efficacy in cells that express P-glycoprotein (Pgp) and the βIII isotype of tubulin, two clinically relevant mechanisms of drug resistance to microtubule targeting agents. The relative resistance (Rr) for the HeLa parental and βIII isotype expressing subline yielded values of 1.0–3.9, values much lower than those obtained with paclitaxel (Table 1). The Pgp-expressing SK-OV-3-MDR-1/M6/6 cells were equally sensitive as the parental SK-OV-3 cells with Rr values of 1.1–1.4. This is in contrast to the Rr value of 240 obtained with paclitaxel. These results show that the Janus compounds have efficacy in a range of cancer cell lines including paclitaxel-resistant cells. 

### 2.5. Effects of Janus Compounds on Mitotic Spindles and Cell Cycle Progression

**Figure 6:**
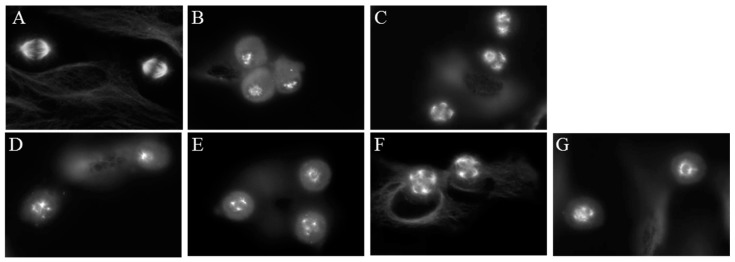
Effects of the Janus compounds on mitotic spindles. HeLa cells were treated for 18 h and then microtubules visualized by indirect immunofluorescence: (**A**) vehicle-treated cells; (**B**,**C**) the effects of the positive controls paclitaxel (100 nM) and CA-4 (5 nM), respectively; and the effects of the Janus compounds: (**D**) 25 µM **1**; (**E**) 25 µM **2**; (**F**) 8 µM **3**; and (**G**) 10 µM **4**.

A well-known property of microtubule binding agents is their ability to disrupt the formation of bipolar mitotic spindles leading to mitotic accumulation. The effects of compounds **1**–**4** on mitotic spindles were visualized using indirect immunofluorescence techniques and cell cycle distribution was evaluated using flow cytometry of HeLa cells following treatment with **1**–**4**. Cells treated with vehicle displayed normal bipolar mitotic spindles (Figure 6A). As expected, paclitaxel and CA-4 caused the formation of multiple mitotic spindle asters (Figure 6B,C). The Janus compounds **1**–**4** also initiated the formation of abnormal mitotic spindles, with multipolar asters observed at concentrations that disrupted interphase microtubules. (Figure 6D–G).

**Figure 7:**
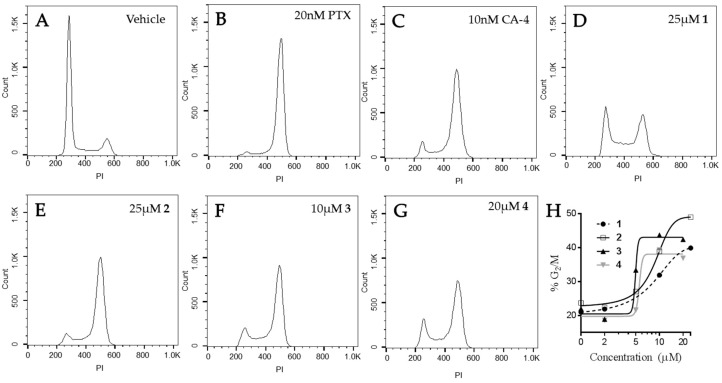
Effects of the Janus compounds on cell cycle distribution. HeLa cells were treated with (**A**) vehicle (DMSO), or (**B**–**G**) the compounds indicated for 18 h and then the DNA was stained with propidium iodide and cell cycle distribution evaluated by flow cytometry. (**H**) The concentration-dependent effects of compounds **1**–**4** on the percentage of cells in G_2_/M.

The effects of the compounds on cell cycle distribution were evaluated (Figure 7). A normal cell cycle distribution with the majority of cells in G_1_ was observed in vehicle-treated HeLa cells. Paclitaxel and CA-4 initiated G_2_/M accumulation of HeLa cells following 18 h of treatment, consistent with the ability of these compounds to cause mitotic spindle defects. The Janus compounds caused concentration-dependent G_2_/M cell cycle accumulation, consistent with the formation of abnormal mitotic spindles shown above in Figure 6. Compounds **2**–**4** showed a classic pattern of G_1_ loss and G_2_/M accumulation similar to paclitaxel and CA-4. Compound **1** caused some accumulation of cells in both the S and G_2_/M phases of the cell cycle, with a concomitant loss of cells in G_1_ at 25 μM. A higher concentration of **1**, 35 µM, did not increase the percentage of cells in G_2_/M indicating that the effects of this compound on microtubules are not sufficient to cause complete mitotic accumulation and suggestive of a second mechanism of cytotoxicity. These data are consistent with the fact that **1** was the least potent in its ability to disrupt cellular microtubules and alter the polymerization of purified tubulin. Although the effects of these compounds on the formation of multipolar spindles and mitotic arrest are included as a mechanism of their antiproliferative effects in cell culture, it is important to note that accumulating evidence indicates that the effects of microtubule targeted agents on interphase signaling are important for their anticancer efficacy [19,20,21,22,23].

### 2.6. Molecular Modeling of Compounds in Colchicine Site of Tubulin

Structurally related tricyclic pyrimido[4,5-*b*]indoles have been previously reported to bind at the colchicine site on tubulin and cause displacement of [^3^H]colchicine [12] and thus compounds **1**–**4** were docked in the X-ray crystal structure of the colchicine binding site of tubulin (PDB ID: 4O2B) [24] using MOE [25]. Colchicine was re-docked into the binding site using the same set of parameters to validate the study. The rmsd of the best docked pose was 0.345 Å, thus validating the docking using MOE.

**Figure 8:**
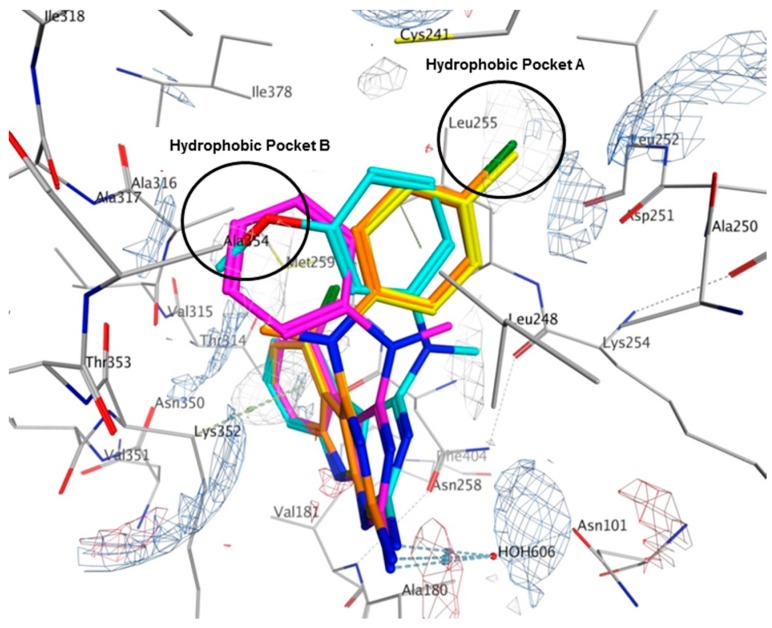
Docked poses of **1** (cyan), **2** (yellow), **3** (magenta) and **4** (orange) in the colchicine site of tubulin (PDB ID: 4O2B). Electrostatic map with electropositive charges in blue, electronegative charges in red and hydrophobic regions in gray.

Multiple low energy conformations of **1**–**4** were obtained on docking. Figure 8 shows the docked poses of **1** (cyan), **2** (yellow), **3** (magenta) and **4** (orange) in the colchicine site of tubulin. The pyrimido[4,5-*b*]indole scaffold of **1**–**4** forms hydrophobic interactions with Alaα180, Valα181, Leuβ248, Asnβ258, Metβ259, Thrβ314 and Lysβ352. The 2-NH_2_ group undergoes hydrogen bonding with HOH606. The N4-Me in **1** and **3** lies in a pocket lined by hydrophobic residues Alaβ250 and Lysβ254. However, in **2** and **4**, the N4-Me undergoes hydrophobic interaction with Alaβ354 and side chain alkyl groups on Lysβ352. The N4-aryl substitution in **1**, **2** and **4** lies in a larger hydrophobic pocket (Hydrophobic Pocket A, Figure 8) lined by residues Cysβ241, Leuβ248, Alaβ250 and Leuβ255. In **3**, the anilino group adopts an alternate conformation where it interacts with residues Cysβ241, Leuβ248, Ileβ318, Lysβ352 and Alaβ354 in the smaller hydrophobic pocket (Figure 8, Hydrophobic Pocket B).

**Figure 9:**
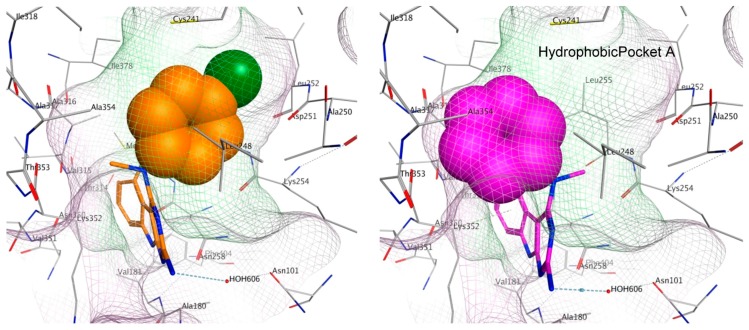
Space fill view of the docked poses of **4** (orange) and **3** (magenta) in the colchicine binding site on tubulin. Hydrophobic surface is in green and hydrophilic surface is in pink.

To elucidate the loss in the depolymerizing activity of **3** compared to **4**, a comparison of the space fill view of the docked pose of **3** and **4** in the binding site was performed. In **4**, the aryl ring lies in a hydrophobic pocket (shaded in green, Figure 9). However, in **3**, the unsubstituted phenyl ring lacking any hydrophobic group on the ring is unable to orient itself towards the hydrophobic pocket A and occupies a partially hydrophilic pocket (shaded in pink), which could result in an unfavorable conformation. As a consequence, binding of **3** at the colchicine site could lower the tubulin destabilizing activity as observed in the immunofluorescence and tubulin polymerization experiments. For the best docked pose of **1**–**4**, the scores were −6.31, −6.18, −6.04 and −6.13 kcal/mol, respectively.

## 3. Discussion

Our laboratories have focused on the screening and biological evaluation of synthetically derived compounds that bind to tubulin within the colchicine site. In the course of evaluating a large library of compounds, the four compounds (**1***–***4**) described here are unique in their ability to cause depolymerizing effects (like classical colchicine site binding compounds) and also cause the formation of microtubule bundles, reminiscent of microtubule stabilizers. Our studies suggest that these compounds have unique, dual effects on microtubules and on purified tubulin in that they have properties of both classical microtubule destabilizing and stabilizing compounds.

In our evaluations of the cellular and biochemical effects of these four compounds, we found that they lie on a continuum with regard to their effects on microtubule structure. Compound **2** has the most effective microtubule destabilizing activity as demonstrated by a dose-dependent inhibition of tubulin polymerization, clear destabilizing effects in cells, and the ability to initiate G_2_/M arrest. Importantly, this was the only Janus compound for which inhibition of tubulin polymerization was observed at any concentration. In contrast, compound **3** has the most potent and effective microtubule stabilizing activity as observed by its sole ability to increase the initial rate of tubulin polymerization at sub-stoichiometric concentrations. The finding that **3** has only modest effects on microtubule depolymerization with predominant microtubule stabilizing effects is consistent and partly rationalized with the modeling studies showing that **3** fails to fully occupy the hydrophobic pocket of the colchicine site leading to unfavorable interactions within this classical destabilizer site. Compounds **1** and **4** have clear microtubule destabilizing effects and mixed effects at higher concentrations, yet neither affected tubulin polymerization at stoichiometric concentrations or caused full G_2_/M arrest at concentrations that elicited microtubule disruption. These results suggest that they have the weakest microtubule effects of this series. We suggest that their antiproliferative activities in cancer cells could be due to additional biological targets as has been previously observed for other small molecules that interact with the colchicine site [14]. Thus, the subtle chemical differences among these compounds allows them each to interact with tubulin to elicit a mixture of stabilizing and destabilizing effects, but they are remarkable in their distinct propensities to initiate these competing actions.

Although these compounds have characteristics that can be classified as microtubule stabilizing and destabilizing actions, they are notably distinct from the effects of classical stabilizing and destabilizing drugs. The initial microtubule depolymerizing effects of **1**, **2**, and **4** are consistent with the effects of other colchicine site agents, including CA-4, at low concentrations. However, they are different from classic destabilizers because at higher concentrations they cause the formation of rigid, bundled microtubules. While the destabilizing effects of these compounds are not completely reminiscent of classic colchicine site binding agents, their microtubule stabilizing effects are also distinct from those seen with paclitaxel. Compound **3** has the strongest propensity for microtubule stabilization, but **3** also initiated microtubules with a less rigid and more disorganized morphology at lower concentrations and then, at higher concentration, the formation of microtubule bundles that are more needle-like than those initiated by paclitaxel. Furthermore, while **3** increases the rate of initial purified tubulin polymerization, it does not increase the extent of total tubulin polymer to the degree as paclitaxel even when present at five-fold higher concentrations. Therefore, although **3** has demonstrated microtubule stabilizing activity both in cells and with purified tubulin, its effects are distinct from classic taxane-site microtubule stabilizing drugs. Together, these findings demonstrate that the mixed effects of the Janus compounds are distinct from the effects of classical microtubule stabilizing or destabilizing agents.

In summary, the Janus compounds **1***–***4** provide new chemical small molecule probes that can alter the balance between tubulin heterodimer and microtubule polymer in a concentration dependent manner. It will be valuable in future studies to identify whether occupancy within a specific orientation within the colchicine site can initiate microtubule stabilizing effects or if these compounds have the ability to bind to and stabilize microtubules by binding to an additional site on tubulin/microtubules. These studies will be complex since joint incubations of the Janus compounds with microtubule stabilizing or destabilizing drugs will, by definition, alter the tubulin/microtubule balance. Colchicine site agents bind to tubulin heterodimers and microtubule stabilizers bind to tubulin polymer. Thus, a Janus compound will affect both the direct competitive binding of these compounds and shift the tubulin heterodimer/microtubule balance to impact binding at a non-competitive level. Regardless of whether the Janus compounds elicit microtubule stabilization by binding in a unique pose in the colchicine site, by binding in a known stabilizer site, or through interacting with a previously uncharacterized site on tubulin, the finding that a synthetically tractable, small molecule can elicit microtubule stabilizing effects that have exclusively been attributed to large natural products is a significant finding. The Janus compounds have the potential to alter the current paradigm of how microtubule stabilization can occur.

## 4. Materials and Methods

### 4.1. Chemistry

All evaporations were carried out in vacuum with a rotary evaporator. Analytical samples were dried in vacuo (0.2 mm Hg) in a CHEM-DRY drying apparatus over P_2_O_5_ at 50 °C. Thin–layer chromatography (TLC) was performed on Whatman^®^ Sil G/UV254 silica gel plates (GE Healthcare Bio-Sciences, Pittsburgh, PA, USA), and the spots were visualized by irradiation with ultraviolet light (254 and 366 nm). Proportions of solvents used for TLC are by volume. All analytical samples were homogeneous on TLC in at least two different solvent systems. Column chromatography was performed on a 70−230 mesh silica gel (Fisher Scientific, Pittsburgh, PA, USA) column. The amount (weight) of silica gel for column chromatography was in the range of 50−100 times the amount (weight) of the crude compounds being separated. Columns were wet-packed with appropriate solvent unless specified otherwise. Melting points were determined using an MPA100 OptiMelt automated melting point system (Stanford Research Systems Inc., Sunnyvale, CA, USA), and are uncorrected. Nuclear magnetic resonance spectra for protons (^1^H-NMR) were recorded on Bruker Avance II 400 (400 MHz) and 500 (500 MHz) systems (Bruker Daltonics Inc., Billerica, MA, USA), and were analyzed using MestReC NMR data processing software (Version 4.8.1.1, Mestrelab Research, Escondido, CA, USA). The chemical shift (δ) values are expressed in ppm (parts per million) relative to tetramethylsilane as an internal standard: s, singlet; d, doublet; t, triplet; q, quartet; m, multiplet; br, broad singlet; exch, protons exchangeable by addition of D_2_O.

Elemental analyses were used to determine the purities of the target compounds. Elemental analyses were performed by Atlantic Microlab, Inc. (Norcross, GA, USA). Elemental compositions are within ±0.4% of the calculated values and indicate >95% purity. Fractional moles of water or organic solvents frequently found in some analytical samples could not be prevented despite 24–48 h of drying in vacuo and were confirmed where possible by their presence in the ^1^H-NMR spectra. All solvents and chemicals were purchased from Sigma-Aldrich Co. (St. Louis, MO, USA) or Fisher Scientific Inc. and were used as received.

*N-(5-Chloro-4-oxo-4,9-dihydro-3H-pyrimido[4,5-b]indol-2-yl)-2,2-dimethyl propanamide* (**6**). Compound **5** (2 g, 8.5 mmol) and 150 mL of pivalic anhydride were taken in a 250 mL round bottomed flask. The reaction mixture was stirred at 120 °C for 2.5 h. Then, hexane was added at room temperature, which resulted in precipitation of a pale brown solid. The precipitate was filtered and washed with hexane to afford 1.6 g (60%) of **6** as a brown solid. TLC *R*_f_ = 0.80 (CHCl_3_/MeOH, 5:1); m.p. 189.2–189.8 °C [16] (185.8–190.1 °C). ^1^H-NMR (500 MHz, DMSO-*d*_6_): δ = 1.26 (s, 9H, C(CH_3_)_3_), 7.19–7.37 (m, 3H, Ar), 11.16 (s, 1H, 2-NH, exch), 11.93 (s, 1H, 3-NH, exch), 12.11 (s, 1H, 9-NH, exch). ^1^H-NMR agreed well with the previously reported values [16].

*N-(4,5-Dichloro-9H-pyrimido[4,5-b]indol-2-yl)-2,2-dimethyl propanamide* (**7**). Compound **6** (1 g, 3.137 mmol) was treated with 200 mL of POCl_3_ in a 500 mL round bottom flask. The reaction mixture was heated to reflux for 4 h. The POCl_3_ was evaporated and the mixture was neutralized using conc. NH_4_OH solution (28 wt %–30 wt % in water). The aqueous mixture was filtered (the precipitate being the compound) and the precipitate was dried and dissolved in chloroform. To the solution was added silica gel (3 g) and solvent was removed under reduced pressure to provide a plug, which was purified by column chromatography using chloroform and 1% methanol in chloroform. Fractions containing the product (TLC) were pooled and evaporated to provide 710 mg (67%) of **7** as a brown solid. TLC *R*_f_ = 0.86 (CHCl_3_/MeOH, 5:1); m.p. 245.9–246.5 °C (245.6–246.1 °C). ^1^H-NMR (500 MHz, DMSO-*d*_6_): δ = 1.24 (s, 9H, C(CH_3_)_3_), 7.36–7.50 (m, 3H, Ar), 10.30 (s, 1H, 9-NH, exch), 12.96 (s, 1H, 2-NH, exch). ^1^H-NMR was consistent with the reported [16] values.

### 4.2. General Procedure for the Synthesis of 5-Chloro-N^4^-methyl-N^4^-aryl-9H-pyrimido [4,5-b]indole-2,4-diamines ***1**–**4***

To a solution of **7** (1 equivalent) in 40 mL of *i*-propanol was added *N*-methyl anilines **8**–**11** and 2 drops of conc. HCl. The reaction mixture was heated to reflux for 70 h, cooled to room temperature and then neutralized with 4 mL of 1 N NaOH solution. The reaction was then heated to reflux for 2 h. The solvent was then evaporated to obtain a dark brown colored solid. The resulting precipitate was then dissolved in chloroform and methanol. To the solution was added silica gel, four times the weight of the reaction mixture, and the solvent was removed under reduced pressure to provide a plug. The plug was purified by column chromatography using 1% methanol in chloroform. Fractions containing the product (TLC) were pooled and evaporated to give a solid which was further purified by washing with hexane to give target compounds **1**–**4** in 60%–67% yield.

*5-Chloro-N^4^-(3-methoxyphenyl)-N^4^-methyl-9H-pyrimido[4,5-b]indole-2,4-diamine* (**1**). Using the general procedure described above, compound **7** (100 mg, 0.30 mmol) was treated with 3-methoxy-N-methyl aniline **8** (122 mg, 0.90 mmol) to give 65 mg (62%) of **1** as a brown solid. TLC R_f_ = 0.39 (CHCl_3_/MeOH, 15:1); m.p. 226.8–227.2 °C. ^1^H-NMR (400 MHz, DMSO-*d*_6_): δ = 3.34 (s, 3H, OCH_3_), 3.59 (s, 3H, NCH_3_), 6.28–6.38 (m, 3H, Ar), 6.63 (s, 2H, NH_2_, exch), 7.00–7.05 (m, 2H, Ar), 7.18–7.22 (m, 1H, Ar); 7.28–7.30 (m, 1H, Ar); 11.82 (s, 1H, 9-NH, exch). Elemental analysis calculated (%) for C_18_H_16_ClN_5_O∙0.23CH_3_COCH_3_: C, 61.14; H, 4.77; N, 19.07; Cl, 9.66. Found: C, 60.88; H, 4.57; N, 19.14; Cl, 9.38.

*5-Chloro-N^4^-methyl-N^4^-(p-tolyl)-9H-pyrimido[4,5-b]indole-2,4-diamine* (**2**). Using the general procedure described above, compound **7** (100 mg, 0.30 mmol) was treated with *N*-methyl-*p*-toluidine **9** (108 mg, 0.90 mmol) to provide 60 mg (60%) of **2** as a brown solid. TLC *R*_f_ = 0.38 (CHCl_3_/MeOH, 15:1); m.p. 231.9–232.8 °C. ^1^H-NMR (400 MHz, DMSO-*d*_6_): δ = 2.19 (s, 3H, CH_3_), 3.32 (s, 3H, NCH_3_), 6.53 (s, 2H, NH_2_, exch), 6.70–6.72 (m, 2H, Ar), 6.94–6.96 (m, 2H, Ar), 7.00–7.02 (m, 1H, Ar), 7.16–7.19 (m, 1H, Ar), 7.25–7.27 (m, 1H, Ar), 11.74 (s, 1H, 9-NH, exch). Elemental analysis calculated (%) for C_18_H_16_ClN_5_: C, 63.99; H, 4.77; N, 20.73; Cl, 10.49. Found: C, 63.85; H, 4.83; N, 20.44; Cl, 10.56.

*5-Chloro-N^4^-methyl-N^4^-phenyl-9H-pyrimido[4,5-b]indole-2,4-diamine* (**3**). Using the general procedure described above, compound **7** (100 mg, 0.30 mmol) was treated with *N*-methyl aniline **10** (95 mg, 0.90 mmol) to afford 64 mg (67%) of **3** as a brown solid. TLC *R*_f_ = 0.42 (CHCl_3_/MeOH, 15:1); m.p. 228.1–229.1 °C. ^1^H-NMR (400 MHz, DMSO-*d*_6_): δ = 3.34 (s, 3H, NCH_3_), 6.61 (s, 2H, NH_2_, exch), 6.76–6.80 (m, 3H, Ar), 7.02–7.04 (m, 1H, Ar), 7.12–7.21 (m, 3H, Ar), 7.27–7.29 (m, 1H, Ar), 11.81 (s, 1H, 9-NH, exch). Elemental analysis calculated (%) for C_17_H_14_ClN_5_: C, 63.06; H, 4.36; N, 21.63; Cl, 10.95. Found: C, 63.06; H, 4.25; N, 21.62; Cl, 10.86.

*5-Chloro-N^4^-(4-chlorophenyl)-N^4^-methyl-9H-pyrimido[4,5-b]indole-2,4-diamine* (**4**). Using the general procedure described above, compound **7** (100 mg, 0.30 mmol) was treated with 4-chloro-*N*-methyl aniline **11** (126 mg, 0.90 mmol) to afford 68 mg (64%) of **4** as a brown solid. TLC *R*_f_ = 0.39 (CHCl_3_/MeOH, 15:1); m.p. 226.4–227.2 °C. ^1^H-NMR (400 MHz, DMSO-*d*_6_): δ = 3.35 (s, 3H, NCH_3_), 6.66 (s, 2H, NH_2_, exch), 6.75–6.77 (m, 2H, Ar), 7.04–7.06 (m, 1H, Ar), 7.16–7.19 (m, 3H, Ar), 7.28–7.30 (m, 1H, Ar), 11.86 (s, 1H, 9-NH, exch). Elemental analysis calculated (%) for C_17_H_13_Cl_2_N_5_∙1.0CH_3_OH: C, 55.40; H, 4.39; N, 17.94; Cl, 18.17. Found: C, 55.34; H, 4.33; N, 18.04; Cl, 18.06.

### 4.3. Molecular Modeling 

Compounds **1**–**4** were docked in the X-ray crystal structure of the colchicine binding site of tubulin (PDB ID: 4O2B, 2.30 Å) [24] using Molecular Operating Environment (MOE2015.10) [25]. Protein was prepared as previously reported [15]. Ligands were sketched using the builder function in MOE and minimized using Amber10:EHT forcefield. The ligands were then docked in the binding site using the default settings in the docking protocol. The placement was performed using Triangle Matcher and scored using London dG. The refinement was carried out using Rigid Receptor and scored using GBVI/WSA dG.

### 4.4. Biological Evaluations

SK-OV-3 and HeLa cells were purchased from ATCC (Manassas, VA, USA). The HeLa WT βIII and SK-OV-3-MDR-1-M6/6 cell lines were previously described [26]. MDA-MB-435 cells were obtained from the Lombardi Cancer Center (Georgetown University, Washington, DC, USA). The HeLa, SK-OV-3, and SK-OV-3-MDR-1-M6/6 cell lines were grown in Basal Medium Eagle, the WTβIII cells were maintained in DMEM and the MDA-MB-435 cell line grown in IMEM (Richter’s Modification). All media were supplemented with 10% fetal bovine serum and 50 µg/mL gentamicin antibiotic other than the MDA-MB-435 cells, which were supplemented with only 25 µg/mL gentamicin. All cell stocks were maintained in liquid nitrogen and all experiments conducted within 6 months of cell retrieval.

### 4.5. Fluorescence Microscopy

HeLa cells were plated onto glass coverslips and allowed to adhere and grow for 24 h. Cells were then treated with the vehicle (DMSO) or compounds for 18 h. The effects on microtubules and mitotic spindles were evaluated by indirect immunofluorescence using a β-tubulin antibody. The DNA was stained using DAPI. A Nikon Eclipse Ti80 microscope with Nikon Advanced Research Imaging Software 3.2 (Melville, NY, USA) was used to acquire and process the images. Each experiment was performed a minimum of three times.

### 4.6. Inhibition of Cellular Proliferation

The sulforhodamine B (SRB) assay was used to evaluate the antiproliferative effects of the compounds as described previously [26]. Cells were treated with the compounds indicated for 48 h then cellular protein fixed and stained with SRB dye. The log concentration-response curves were generated and IC_50_ values calculated by linear regression. Each experiment was conducted a minimum of three times, each in triplicate.

### 4.7. Cell Cycle Analysis

HeLa cells were plated and allowed to adhere and proliferate for 24 h. Cells were treated with compounds for 18 h and harvested on ice. Krishan’s reagent was used to stain the DNA [27]. The cell cycle distribution was evaluated using a BD FACSCalibur (BD Biosciences, San Jose, CA, USA) and data were analyzed using the FlowJo v10.2 single cell analysis software (FlowJo LLC, Ashland, OR, USA). Quantification of the concentration-dependent effects of compounds on G_2_/M accumulation was performed using the Muse Cell Analyzer (Millipore, Billerica, MA, USA).

### 4.8. Tubulin Polymerization and Electron Microscopy 

Compounds were evaluated for their effects on polymerization of tubulin by light scattering and the structure of the microtubule polymer by electron microscopy as previously described [28]. Briefly, purified porcine brain tubulin (Cytoskeleton Inc., Denver, CO, USA) at a concentration of 2 mg/mL was mixed with GPEM buffer (80 mM PIPES pH 6.8, 1 mM MgCl_2_, and 1 mM EGTA) with 1 mM GTP and 10% glycerol. The solution was incubated with compounds as indicated and polymerization followed for 30 min at 37 °C. Aliquots were collected and fixed with 4% glutaraldehyde and then mounted on 200 mesh Cu grids and negatively stained with 8% uranyl acetate. Tubulin polymers were visualized using a JEOL100CX transmission electron microscope (Akishima, Tokyo, Japan) with a magnification range of 2000–100,000×.

## Figures and Tables

**Table 1:** Antiproliferative effects of the Janus compounds **1**–**4**. The effects of the compounds on the inhibition of cancer cell proliferation were evaluated using the SRB assay in MDA-MB-435 cells and in isogenic pairs of cell lines that are sensitive or resistant to paclitaxel, respectively. The WT βIII cell line is a βIII-tubulin expressing subline of HeLa and the SK-OV-3-MDR-1/M6/6 is a P-glycoprotein expressing subline of SK-OV-3 cells. The IC_50_ (concentration that inhibits growth 50%) values were calculated from the linear portions of log dose–response curves. The relative resistance (Rr) was determined by dividing the IC_50_ of the resistant cell line by the IC_50_ of the parental cell line for each isogenic cell line pair. Paclitaxel (PTX) and CA-4 were used as positive controls. *n* = 3 ± SD.

Compound (μM)	MDA-MB-435	HeLa	WT βIII	Rr	SK-OV-3	SK-OV-3-MDR-1/M6/6	Rr
**1**	6.5 ± 0.3	1.4 ± 0.2	5.4 ± 0.2	3.9	5.4 ± 0.8	7.4 ± 1.0	1.4
**2**	3.0 ± 0.3	2.4 ± 0.1	2.7 ± 0.2	1.1	2.5 ± 0.1	3.1 ± 0.2	1.2
**3**	4.2 ± 0.5	2.4 ± 0.4	4.6 ± 0.1	1.9	5.0 ± 0.4	5.4 ± 0.03	1.1
**4**	4.2 ± 0.5	3.5 ± 0.1	3.4 ± 0.1	1.0	4.1 ± 1.0	4.7 ± 0.5	1.1
PTX (nM)	4.5 ± 0.5	2.8 ± 0.4	24 ± 3.0	8.6	5.0 ± 0.6	1200 ± 60	240
CA-4 (nM)	4.4 ± 0.5	3.3 ± 0.4	3.3 ± 0.3	1.0	5.5 ± 0.5	7.2 ± 1.1	1.3
